# Parthenogenesis in dipterans: a genetic perspective

**DOI:** 10.1098/rspb.2023.0261

**Published:** 2023-03-29

**Authors:** A. L. Sperling, D. M. Glover

**Affiliations:** ^1^ Department of Genetics, University of Cambridge, Cambridge, UK; ^2^ California Institute of Technology, Pasadena, CA, USA

**Keywords:** parthenogenesis, reproduction, *Drosophila*, dipterans, development

## Abstract

Parthenogenesis has been documented in almost every phylum of animals, and yet this phenomenon is largely understudied. It has particular importance in dipterans since some parthenogenetic species are also disease vectors and agricultural pests. Here, we present a catalogue of parthenogenetic dipterans, although it is likely that many more remain to be identified, and we discuss how their developmental biology and interactions with diverse environments may be linked to different types of parthenogenetic reproduction. We discuss how the advances in genetics and genomics have identified chromosomal loci associated with parthenogenesis. In particular, a polygenic cause of facultative parthenogenesis has been uncovered in *Drosophila mercatorum,* allowing the corresponding genetic variants to be tested for their ability to promote parthenogenesis in another species, *Drosophila melanogaster*. This study probably identifies just one of many routes that could be followed in the evolution of parthenogenesis. We attempt to account for why the phenomenon has evolved so many times in the dipteran order and why facultative parthenogenesis appears particularly prevalent. We also discuss the significance of coarse genomic changes, including non-disjunction, aneuploidy, and polyploidy and how, together with changes to specific genes, these might relate to both facultative and obligate parthenogenesis in dipterans and other parthenogenetic animals.

## 1. Introduction

Insects are the most abundant and diverse class of animals on Earth and therefore provide excellent models to study reproductive diversity and genome evolution. There are approximately 29 orders of insects of which the true flies (Diptera) are one of the most populous, after beetles (Coleoptera) and butterflies (Lepidoptera). Among the best-known dipterans are the first genetic model organism *Drosophila melanogaster*, the disease vector mosquitos *Anopheles* and *Culex*, and the ubiquitous house fly *Musca domestica*. However, there are approximately 160 000 documented extant species of flies and this is likely to be a great underestimate [1]. Owing in part to their diversity and adaptability, dipterans have evolved multiple different reproductive strategies, from the use of both female homo- and hetero-gamety for sex determination to the use of parthenogenetic forms of reproduction [2,3]. Of the documented species, nearly 150 are currently known to employ parthenogenetic reproduction, a figure likely to be a gross underestimate. Indeed, parthenogenesis has evolved in at least 20 families of flies, and although extremely rare, it can even arise sporadically in *D. melanogaster* [4]*. Drosophila melanogaster* is the most renowned species of *Drosophila,* but there are at least 2000 species in the *Drosophila* genus and the majority of those studied are capable of some degree of parthenogenetic reproduction.

The term ‘parthenogenesis’ was coined during the mid-nineteenth century [5]; its etymology is modern Greek, from *parthenos* meaning ‘virgin’, and *genesis*, meaning ‘origin’ or ‘creation’. However, the phenomenon was first described in aphids by Charles Bonnet in the 1740s [6] and since then has been found to occur in most animal phyla. We provide a parthenogenesis lexicon for the terms we will use as well as an overview of the underlying biology of parthenogenesis in the electronic supplementary material, S1.

**Figure 1.**
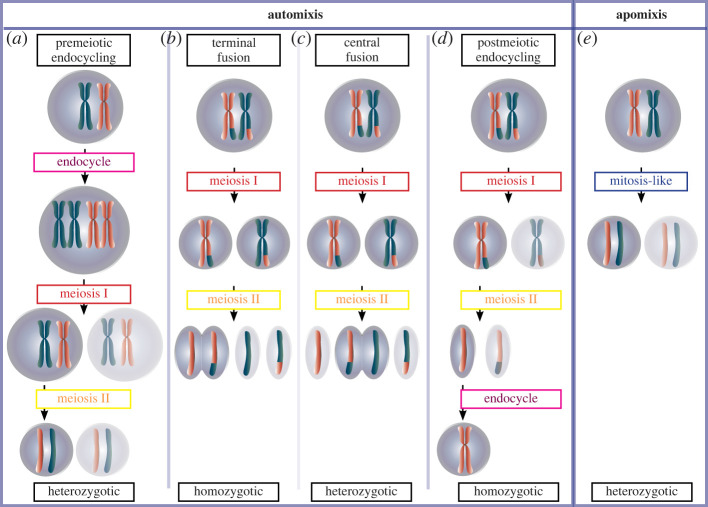
Simplified schematic of the mechanisms of parthenogenetic egg maturation showing metaphase of automictic (*a*–*d*) and apomictic (*e*) parthenogenesis showing rediploidization and zygosity. Both terminal and central fusion are also a representative of a failure in cytokinesis in meiosis II or meiosis I, respectively. Chromosomes, pink and blue; centrosomes, red and yellow; meiotic/mitotic spindle, yellow.

Parthenogenesis requires that embryonic development is initiated in an egg without requiring genomes from two individual parents and therefore any offspring will only have a version of the maternal genome. Other types of non-canonical reproduction, for example, ones that require sperm or even ones where the paternal genome supplants the maternal genome in the egg, are described in the electronic supplementary material, S1. There are two common forms of parthenogenesis, one where the cells skip meiosis and diploidy is retained and another where meiosis occurs and diploidy is restored. When meiosis is retained, the absence of the sperm's haploid nuclear contribution must be compensated for by the generation of diploid cells from one or more of the products of female meiosis (an overview of mitosis and meiosis is given in the electronic supplementary material, figure S1). There are several possible routes for rediploidization that may be taken before, during and after meiosis (figure 1).

In this review, we focus upon parthenogenetic dipterans and discuss their propensity for parthenogenetic reproduction considering aspects of their development and genetics. We also relate observations in parthenogenetic dipterans to the genomic abnormalities that arise in other parthenogenetic animals. We refer the reader to the following cited articles for discussion about barriers to parthenogenesis [7], developmental constraints [8], ecological implications [9], involvement of centriole inheritance/formation [10], and further discussion about parthenogenesis in *Drosophila* [11].

## 2. Dipteran biology lends itself to parthenogenesis

**Figure 2.**
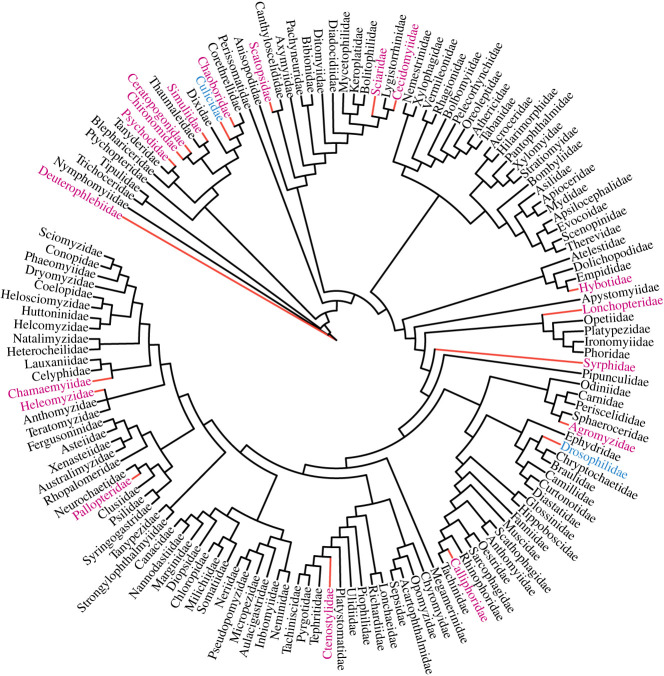
Phylogenetic tree of dipteran families based on multiple nuclear genes [13] with minor alterations [14] visualized using iTOL: interactive tree of life. Families containing parthenogenetic species are indicated with a red branch. The majority obligate parthenogenetic containing families have a pink label and the majority facultative parthenogenetic containing families have a blue label.

In contrast to a theory that parthenogenesis is rare in animals that fly owing to their increased ability to relocate to find a mate [12], parthenogenesis appears to occur across the order Diptera (figure 2) and is more prevalent than originally believed, particularly if one also considers the often-overlooked facultative parthenogenesis. Parthenogenesis has been documented in 20 families and nearly 150 species of flies (table 1; electronic supplementary material, table S1).

**Table 1.** Simplified summary of dipteran species groups that have been documented to have some incidence of parthenogenesis, with the likely type, mechanism, incidence of polyploidy, and important features. (Refer to the electronic supplementary material, table S1 for the detailed list with references.)

parthenogenetic dipterans												
common name	family	facultative			paedogenetic			probably obligate				comments
			auto-		auto-	apo-	polyploid		auto-	apo-	polyploid	
mountain midge	Deuterophlebiidae							1				possible geographical parthenogenesis
drain fly	Psychodidae							4		1	1	two species lost the need for a blood meal
mosquito	Culicidae	4	1									strains are facultative parthenogenetic, possibly many more yet to be identified
phantom midges	Chaoboridae							1				loss of the periodicity to development
blackfly	Simuliidae							4			4	all are triploid and probably sympatric with their parent species
no-see-um	Ceratopogonidae							3				all are able to be maintained as laboratory cultures and are thus good model organisms
non-biting midges	Chironomidae	8		2				53		8	3	a number of these species have had their maturation divisions described
dung midges	Scatopsidae							1				unique in reproductive anatomy (spermatheca)
dark-winged fungus gnat	Sciaridae							1				Sciaridae family are able to select the sex of their offspring through sex chromosome elimination
gall midges	Cecidomyiidae		4		1	3	4	2				Cecidomyiidae have somatic chromosome elimination
dance flies	Hybotidae							5				Hybotidae females have secondary sexual characteristics that could be studied in parthenogenetic strains
spear-winged flies	Lonchopteridae							1	1			sympatric sexual and parthenogenetic strains, clonal diversity
hoverflies	Syrphidae		1									paedogenesis needs to be confirmed
leaf-miner flies	Agromyzidae							2			1	agricultural pests
fruit flies	Drosophilidae	38	7					2	2			nearly all are facultative
blowflies	Calliphoridae		1									paedogenesis needs to be confirmed
none	Ctenostylidae							2				possibly more parthenogenetic species in this family
flutter-wing flies	Pallopteridae							1				
sun flies	Heleomyzidae							1				
silver flies	Chamaemyiidae						1	1				
	totals	49	8	8	1	3	4	85	3	9	10	

### (a) Dipteran development

Several features of dipteran development may give a predisposition to parthenogenesis as a developmental option. Of these, the development of the egg as a syncytium is of particular importance that appears to be found across the order [15]. Not only is meiosis completed in this self-enclosed environment, but subsequent syncytial embryonic development requires repeated division cycles in the common cytoplasm. These features can influence the ability for parthenogenetic reproduction in several ways.

First, fertilization is not required for the completion of meiosis as documented in most *Drosophila* species [16] but instead, eggs are activated during oviposition [17], thus giving unfertilized eggs the potential to initiate embryogenesis.

Second, *Drosophila* can overcome a problem faced by automictic parthenogens, which retain complete meiosis, of how to re-diploidize their genomes following the reductive meiotic division of a diploid genome. Such rediploidization can occur through fusion of the haploid products of meiosis or by genome duplication (figure 1*b–d*). The close proximity of such haploid nuclei in the common syncytium can facilitate their fusion to generate diploid nuclei [15]. Indeed, in the eggs of *D. melanogaster* and *Drosophila subobscura,* three of the four products of female meiosis (the polar body nuclei) naturally fuse together during the development of fertilized eggs [18,19]. Thus, the machinery enabling nuclear fusion is present in the egg and early embryo and could therefore facilitate the parthenogenetic process.

Third, dipterans have an ability to exit the conventional cell cycle and undertake varying degrees of endoreduplication of their genomes, thus offering another route to diploidization. Indeed, in the syncytial embryos of *D. melanogaster,* mutants in any of the three subunits of the PanGu protein kinase enable the activation of eggs and the participation of the four haploid nuclei in varying rounds of division followed by endoreduplication of their DNA [20,21]. It is possible therefore that a marginal shift in the activity of this protein kinase could pave the way towards parthenogenetic reproduction.

Fourth, the dipteran egg is provided with a rich maternal dowry of all proteins and messenger RNAs it requires for the multiple nuclear division cycles that precede cellularization, which in *D. melanogaster* occurs in cycle 14. This maternal dowry provides many cell cycle regulatory molecules, including checkpoint proteins, that monitor the completion of various stages of the cycle before the next is undertaken. Even subtle changes to these regulatory pathways could enable the onset of the embryonic nuclear division cycles to initiate parthenogenetic development. The maternal dowry is of particular importance in fulfilling the requirement for centrioles in early development. In the great majority of animal species, centrioles are provided paternally in the form of the basal body of the sperm, often together with a centriole-like precursor structure [10]. Centrioles form the scaffold that is used to organize maternally provided pericentriolar material into functional centrosomes, which organize microtubules, the major component of the cell division apparatus. Centrioles may also activate development. This is most dramatically seen in the frog *Xenopus laevis,* where parthenogenesis can be initiated simply by the addition of exogenous centrioles into an unfertilized egg [22–24]. Although reported to be dispensable for the development of the adult body of *Drosophila* [25–27], centrioles are essential to organize the rapid rounds of mitosis of the syncytial embryo and for male meiosis [28]. In *D. melanogaster*, all components of the centriole are part of the maternal contribution to the egg and so only need to be assembled in successive division cycles. In dividing diploid tissue, there is a requirement for a mother centriole on which a daughter can form and so duplicate the structure. In the very first division of the *Drosophila* zygote, one centriole is provided by the basal body of the sperm and the other by an immature centriole-like structure associated with the sperm. However, centrioles can be formed de novo in unfertilized eggs without any reliance upon the sperm to provide a centriole scaffold [29] and this process can be triggered by the provision of Polo-like-kinase 4 (Plk4/SAK) [30]. The ability of centrioles to form de novo in parthenogenetically reproducing *Drosophila* seems likely to use this pathway [29]. This absolute requirement for centrosomes and the evidence that they are indeed able to form de novo has led many to suspect that there is a change to a regulator of centriole biogenesis underlying naturally occurring parthenogenesis.

In addition to their ability to endoreduplicate and so amplify the genome in specific cell types, some dipterans exhibit the elimination of whole chromosomes at particular stages of their life cycles. Dipteran gall midges have multiple germline chromosomes but undergo chromosome elimination in somatic tissues [31]. There are six parthenogenetic gall midges [32–37], which have had their maturation divisions studied in detail. However, there is little mechanistic understanding of how chromosome elimination occurs and how parthenogenesis might alter this process. Gall midges are not the only family that undergo chromosome elimination, the fungus gnat family are able to select the sex of their offspring through sex chromosome elimination [38].

There are also other unusual twists to development that have evolved in other dipteran species. One of the most interesting is a form of parthenogenesis termed paedogenesis, in which parthenogenetic offspring develop within the ovaries of larvae, and the offspring exit the mother's body before she reaches adulthood. Nothing is known about how this process occurs in dipterans although there are eight confirmed or potential paedogenic dipterans. These include the hover fly *Eristalis tenax* [39], the blow fly *Calliphora erythrocephala* [40], the gall midges *Heteropeza pygmaea* [33], *Mycophila speyeri* [36] and *Oligarces paradoxus* [37], the non-biting midges *Tanytarsus boiemicus* [41] and *Paratanytarsus grimmii* [42], and the no-see-um *Culicoides bermudensis* [43]. It is of interest to know how paedogenetic ovaries develop in immature larvae and also if it has a similar mechanism in the different families of fly in which it occurs.

Given the variety of dipteran species, it is perhaps not surprising that developmental pathways have been perturbed in a variety of rich ways in the parthenogenetic species. The parthenogenetic phantom midge, *Chaoborus anomalus* [44], has lost the monthly developmental periodicity typical of the sexually reproducing strains, possibly reflecting loss of the need to coordinate hatching in order to have males and females emerge at the same time. The parthenogenetic dung midge, *Anapausis baueri* [45], has much longer spermatheca than other members of this genus and it would be interesting to determine if this anatomical change results in an inability to retain sperm and if it is related to parthenogenetic ability. Perhaps the most striking change in development is seen in the parthenogenetic drain/sand flies *Lutzomyia maruaga* and *Lutzomyia mamedei* [46,47], which are important human disease vectors. Unlike their sexually reproducing counterparts, both species produce eggs without a blood meal, indicating a substantial switch to their innate biology. It will be important to understand the mechanism of this switch in their feeding behaviour; whether it is tied to their parthenogenetic ability and whether it is permanent or possibly a way to pause the requirement of a blood meal in the absence of a food source.

### (b) Environmental adaptations in parthenogenetic dipterans

It is not only the developmental biology of dipterans that facilitates parthenogenesis but also the diversity of the ways in which dipteran species are able to adapt to their wide-ranging environments. Of dipterans other than the drosophilids, parthenogenesis has been studied in the most detail in the spear-winged fly *Lonchoptera bifurcata,* an excellent model due to its broad distribution with both sexually reproducing and parthenogenetic populations. *Lonchoptera bifurcata* is parthenogenetic in the Americas, Hawaii and New Zealand but sexually reproducing in England and Europe [48]. Parthenogenetic *L. bifurcata* strains show genetic variation in clonal populations that are sympatric and vary in proportion according to the season [49,50]. It would be interesting to discover whether the cause of this variation could be pinned down through the sequencing of the genomes of different populations of *L. bifurcata*.

Insect disease vectors also have very specific environmental demands, namely a reliance on a source of a blood meal, often a prerequirement for successful oogenesis. There are four species of facultative parthenogenetic mosquitos, whose ability to undertake parthenogenetic reproduction was discovered when attempting hybridization experiments with another species [51–56]. There are also three species of parthenogenetic no-see-ums, also known as biting-midges, they are blood feeders and thus also potential disease vectors [43,57]. In the case of the sandflies *Lu. maruaga* and *Lu. mamedei*, it appears unlikely that parthenogenesis contributes to the spread of pathogens, since they have lost their requirement for a blood meal upon becoming parthenogenetic. However, facultatively parthenogenetic dipterans do retain their requirement for a blood meal. Thus, facultative parthenogenesis could provide reproductive assurance thereby enabling disease vectors to thrive, even when males may not be present, without a substantial change to their innate biology.

Dipterans have also adapted to the agricultural environment thus becoming agricultural pests. There are four known dipteran agricultural pest species that are parthenogenetic, including a non-biting midge, a gall midge and two species of leaf-miner flies [58–60]. There is little information on the two leaf-miner flies and if their parthenogenetic ability is related to them being a pest. The non-biting midge, *Bryophaenoclaudius furcatus*, is a parthenogenetic species present in the Northern Hemisphere whose diverse diet means that it cannot be easily controlled by switching crops [61]. The paedogenetic gall midge, *H. pygmaea*, is a problematic pest for mushroom cultivators [33,34]. It has been suggested that parthenogenetic animals may be particularly apt at exploiting the controlled environment of greenhouses [62], thus *H. pygmaea* is an excellent example to study if greenhouses attract parthenogens or if they somehow select for them.

## 3. Facultative parthenogenesis in *Drosophila* and other dipterans

Of the drosophilids, only one, *Drosophila mangabeirai* is a fully obligate parthenogen [63]. *Drosophila mercatorum* strains can behave as obligate parthenogens, which has been found with one such strain collected in Hawaii [64] or they may show varying degrees of facultative parthenogenesis [64–66]. Facultative parthenogenesis was first observed in *Drosophila* through failed intraspecies hybridization experiments [4] and since then, some 50 *Drosophila* species have been tested by sexual isolation and the majority found capable of some degree of facultative parthenogenesis in laboratory cultures of unmated females (table 2; electronic supplementary material, table S2). If we were to extrapolate the prevalence of facultative parthenogenesis in *Drosophila* to other dipteran species, this might predict that as many as 76% of dipterans could be capable of some level of sporadic facultative parthenogenesis. However, it is important to note that the propensity for facultative parthenogenesis in *Drosophila* is strain specific and reliant upon the original geographical collection point [4,65–67]. This is likely to be the case for other dipterans. In addition to the many species that show strain-by-strain variability in their parthenogenetic ability, parthenogenesis can also arise stochastically [4]. It is therefore important to be cautious in evaluating such estimates because it is very difficult to conclude whether a dipteran species is or is not capable of facultative parthenogenesis without testing every population.

**Table 2.** Summary of parthenogenetic *Drosophila* species groups, refer to the electronic supplementary material, table S2 for the detailed list with references.

	parthenogenetic *Drosophila*						
	sub-genus	sub-group	not parthenogenetic	rare gynogenetic	facultative	obligate	
	*Sophophora*	*melanogaster*	2	1^a^	4^a^		
		*obscura*			1		
		*willistoni*	4		2	1	
	*Drosophila*	*cardini*			7		
		*funebris*	1		1		
		*melanica*			2		
		*robusta*			1		
		*immigrans*			2		
		*tripunctata*			2		
		*virilis*			1		
		*quinaria*			2		
		*testacea*			1		
		*repleta*	1		9^a^	1^a^	
	*Hirtodrosophila*				1		
	*Scaptodrosophila*		2		1		
	*Scaptomyza*		1		1		
	*Zaprionus*	1					
					
	totals		12	1	38	2	

^a^The same species is placed in the two marked categories.

Although most non-*Drosophila* parthenogenetic dipterans are documented to be obligate (table 1), it should be noted that the non-biting midges and mosquitoes show a similar propensity for facultative parthenogenesis as *Drosophila* [43,51–57,68]. The apparent low occurrence of facultative parthenogenesis outside these species may not be a biological reality but could rather represent a bias in testing because the laboratory culture of most dipteran species has either not been established or is extremely difficult. Moreover, the traditional documentation of parthenogenesis in dipterans through the observation of a skewed sex ratio in collections from the wild, which may misidentify other phenomena as parthenogenesis, would also bias the reporting of obligate parthenogens in non-model organism dipterans. Thus, we suspect that the ability to undertake facultative parthenogenesis may be much higher than reported.

## 4. Genetics and genomics of parthenogenesis

The diversity in types of parthenogenesis and the aspects of biology that permit it to arise strongly suggest it to have an underlying genetic mechanism. However, the genetic mechanism behind any type of parthenogenesis is likely to be species-specific, reflecting aspects of the biology of the species in question.

Until recently, no parthenogenetic dipteran had its genome sequenced [64], however, it was possible to make inferences about multiple possible molecular mechanisms from changes in many sequenced obligate parthenogenetic genomes (of varying quality) including: springtail [69], stick insects [70], aphids [71–73], ant [74], honeybee [75], wasps [76–79], mite [80], tick [81], crayfish [82,83], water flea [84,85], seed shrimps [86], nematodes [87–94], rotifers [95,96], tardigrades [97–99], snail [100], and fish [101]. We will discuss some of these selected genomes below and a detailed investigation into many of the above genomes can be found elsewhere [102]. The crayfish showed the loss of key meiotic genes [82]. The parthenogenetic nematode, *Diploscapter pachys*, skips meiosis I owing to the loss of genes required exclusively for this division [89]. Another parthenogenetic nematode, *Diploscapter coronatus*, is missing genes required for recombination and meiosis [87]. Meiotic genes also show copy number variation in the parthenogenetic Amazon molly, a fish of hybrid origin, leading the authors to propose this may prevent meiosis I [101] even though the genes present were otherwise normal. Finally, in bdelloid rotifers, one of the most ancient of known parthenogens, there is an expansion of genes that may be related to the parthenogenetic lifestyle and expansion of genes for meiotic recombination/DNA repair [95,96]. The latter event may seem counterintuitive since the DNA repair genes are often involved in cell cycle checkpoints and thus increasing their level should prevent parthenogenesis. However, in ancient parthenogens, the above-mentioned expansion of meiotic recombination/DNA repair genes in the genome may provide a ‘work-around’ for the generally reduced levels of DNA repair and recombination. Together these studies indicate that the underlying genetic causes of parthenogenesis may lie in changes to one or more genes that function in recombination or in the meiotic cell cycle but that there is considerable genetic variability in the genomes of parthenogenetic animals. The difficulty in interpreting any of these studies is that they do not include any functional validation of the genes identified and so it is impossible to know if the reported changes are a cause or consequence of parthenogenesis.

There are, however, a handful of studies where likely causative genetic changes have been identified. A locus involved in the transition from facultative to obligate parthenogenesis in *Daphnia* has been identified [84] and a genetic cause behind facultative parthenogenesis has been mapped to the second chromosome of *Drosophila ananassae* [103]. A study of obligate parthenogenesis in the cape honeybee [104] has identified a candidate gene that may be responsible for this phenomenon. In this work, a thelytokous queen (*th*/*th*) was crossed with arrhenotokous sperm (*Ar*) of a closely related species to generate an F_1_ hybrid queen (*Ar*/th) which was then backcrossed to thelytokous males (*th*/*th*). This resulted in the expected segregation of 50% thelytokous females (*th*/*th*) and 50% arrhenotokous females (*Ar*/*th*). The authors then tracked genetic markers to determine which of 12 candidate genes might follow this same pattern of inheritance of the thelytokous trait. This identified a single protein coding gene, whose expression is downregulated in the ovaries of thelytokous females, and which has specific allelic changes associated with known cases of thelytoky but is absent from other sequenced honeybees. The gene they identified is present in bee and ant species but appears not to be present in lepidopteran and dipteran genomes. It encodes a protein in the structural maintenance of chromosomes (SMC) family, whose members participate in generating chromosome integrity, segregation and sister chromatid cohesion. It is, however, unclear how the deficiency of a protein that regulates chromosome structure and segregation would lead to the reported cytological defect in thelytokous females, namely the fusion of the two central pronuclei after meiosis II. This raises the possibilities that the generation of diploid eggs is not the result of pronuclear fusion but owing to a failure of chromatid segregation in meiosis II. Alternatively, another gene could be responsible for thelytoky in this species. In this light, it should be noted that the authors also identified a second gene for a non-coding RNA (ncRNA) that has single nucleotide polymorphisms which track with thelytoky. As ncRNAs can regulate coordinated patterns of expression of several genes, it may be necessary to exercise some caution before assigning the cause of thelytoky to a single gene in this species.

It will be key to test genetic changes that result in parthenogenesis to definitively identify the gene responsible. If possible, candidate genes could be tested through a series of out- and back-crosses to quantitively determine if the females retain their full parthenogenetic ability. An alternative approach would be to alter this gene in the parthenogenetic animal and see if parthenogenesis is lost. The specific change could also be engineered into a closely related sexually reproducing species to determine if this confers parthenogenesis. The only animal in which a genetic cause has been identified and tested in another non-parthenogenetic animal is in the facultative parthenogen *D. mercatorum* and the usually non-parthenogenetic *D. melanogaster* [64]. Facultative parthenogenesis in *D. mercatorum* is polygenic and can probably be caused by several genes. In this case, increased expression of the cell cycle regulatory genes *polo* and *Myc* together with reduced function of the metabolic gene *Desat2* could lead *D. melanogaster* to show comparable levels of facultative parthenogenetic development as seen in *D. mercatorum*. Polo is a protein kinase that regulates a broad set of functions enabling cell cycle progression including the conversion of centrioles into functional centrosomes able to nucleate mitotic microtubules [105]; Myc is a transcription factor that regulates growth and proliferation [106]; and Desat2 is a stearoyl-CoA 9-desaturase, predicted to be involved in unsaturated fatty acid biosynthesis [64]. These studies were possible using the huge selection of genetic tools in the long-established model organism, *D. melanogaster.* However, now that more genomes are being sequenced and as genome manipulation tools become mainstream and affordable, we anticipate that studies of this type will be forthcoming in non-model organisms.

## 5. Chromosome changes in parthenogens

### (a) Non-disjunction and the occurrence of rare males in thelytoky (female producing) parthenogens

**Figure 3.**
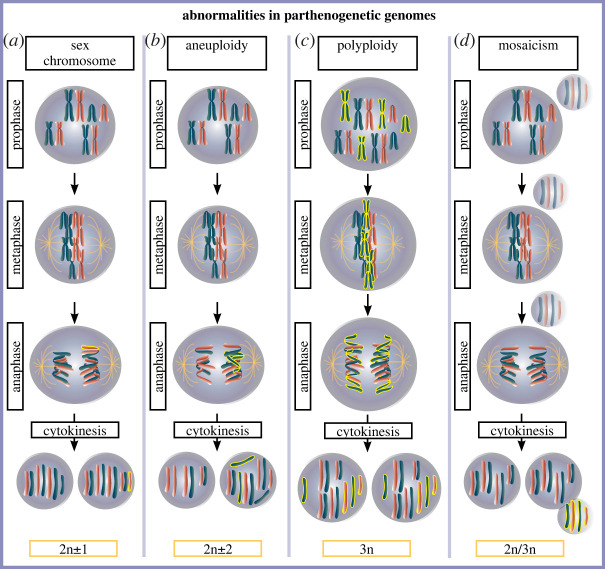
Simplified schematic of the common abnormalities that occur in the genomes of parthenogenetic animals and the ploidy of the resulting animal showing (*a*), non-disjunction; (*b*), aneuploidy; (*c*), polyploidy (specifically triploidy); and (*d*), mosaicism. Chromosome homologues, pink and blue; aberrantly behaving chromosomes, highlighted in yellow; centrosomes, red and yellow; meiotic/mitotic spindle, yellow.

A broad range of gross chromosomal abnormalities have been described to arise in parthenogenetic animals. In addition to local genetic changes, there are genomic changes that appear to be relatively common in parthenogenetic *Drosophila* strains showing facultative parthenogenesis, particularly in the nondisjunction of sex chromosomes during meiosis or during one of the early cleavage divisions (figure 3*a*). Sex-chromosome nondisjunction in *Drosophila* can result in the production of flies that possess one X chromosome and no Y chromosome (X/0), which develop as males because of the heterogametic sex determination system*.* Such X/0 males are sterile because they lack key Y chromosome genes. Dipterans have several different systems for sex determination [2], and so the production of X/0 males cannot be extrapolated to parthenogenesis in other Diptera or other animals in general. Nevertheless, the production of males during thelytoky has been documented in several animals, including grouse locusts [107], brine shrimp [5], isopods [5], mayflies [108], snails [12], fishes [109] and lizards [110]. This phenomenon can be a consequence of nondisjunction and it can also be caused by mosaicism or polyploidy. Non-disjunction resulting in males is well documented in parthenogenetic *Drosophila*, where X/0 males have been recovered in *Drosophila parthenogenetica* [4], *D. mangabeirai* [63], *D. mercatorum* [65], *D. ananassae* [103], *Drosophila pallidosa* [103], and even produced gynogenetically in *D. melanogaster* [111]. In dipterans, the generation of males by nondisjunction can also occur in obligate parthenogenesis but has only been documented in the triploid obligate parthenogenetic silver fly, *Ochthiphila polystigma* [112]. Despite the generation of males through nondisjunction in parthenogenetic dipterans, they do not appear to have any benefit to the parthenogenetic females; females that were ‘mated’ with these sterile X/0 males did not show an increase in the rate of parthenogenesis, at least in *D. parthenogenetica* [4]. Although the generation of X/0 males is a true reproductive dead-end, their occurrence does provide a barometer for the degree of chromosome segregation errors during parthenogenesis.

There are two other types of parthenogenesis that produce males, arrhenotoky and deuterotoky. Arrhenotoky is not possible in most dipterans but was suspected in the gall midge *H. pygmaea* [34]; however, it is more likely that the males undergo chromosome reduction during the post-meiotic divisions until they are haploid (discussed above). It is also possible to produce functional males through deuterotoky. Very little has been documented about this phenomenon in Diptera and it is thought to only occur in the gall midge, *O. paradoxus* [37]. It seems possible that facultative deuterotoky might occur in the mosquito *Aedes aegypti* [51–53], where it has been documented several times, but each time dismissed because both males and females were produced. To summarize: although there is huge variation in the sex-determining systems of dipterans, most parthenogenetic dipterans examined, thus far, fail to produce fertile males.

#### (i) Aneuploidy in parthenogenesis

Non-disjunction resulting in aneuploidy in parthenogens is not restricted to the sex chromosomes but can also affect autosomes. Aneuploidy most frequently arises owing to problems in chromosome pairing during meiosis [113] but can also occur in the mitotic divisions of early development (figure 3*b*). Variation in chromosome number has also been observed at a population level as well as at an individual level. Variations in chromosome number can arise through either nondisjunction or Robertsonian translocations (chromosome arm rearrangements) in parthenogenetic flies [35,37,114,115], cockroaches [116–118], ladybirds [119], aphids [120,121], grasshoppers [122], stick insects [5,12], moths [12], nematodes [123] and planarians [124]. Aneuploidy and chromosome number variation also arise frequently in sexually reproducing populations of animals, including fruit flies [125], beetles [126], mantids [127], fishes [128–131], birds [132], rodents [133–140] and marsupials [141]. The frequency of aneuploidy and chromosome number variation is, however, far greater in parthenogenetic animals. Aneuploidy has also been thought to arise during parthenogenesis because of hybridization between closely related species owing to incompatibilities in their genomes [12]. However, in dipterans, most of the aneuploid species are also gall midges which have chromosome elimination and therefore it could be the result of lagging chromosomes. It is clear that documented aneuploidy is more common amongst the parthenogenetic dipteran species [35,37,114,115] compared to the sexually reproducing dipteran species [125]. Despite this connection between aneuploidy and parthenogenesis, to our knowledge, no studies have been carried out on aneuploidy in the tissues of these parthenogenetic animals.

#### (ii) Polyploidy in parthenogenesis

True-breeding polyploidy (figure 3*c*) is rare in sexually reproducing animals [142], although at least one species of fish [142], salamander [143], frog [144], turtle [145] and rodent [146] have been reported to exhibit polyploidy in natural populations. The low occurrence of polyploidy probably reflects difficulties in chromosome pairing during meiosis or, in some cases, sex chromosome dosage compensation when more than two copies of the genome are present [6,113,142,147,148]. By contrast, changes in ploidy often occur during the transition from sexual to parthenogenetic reproduction in parthenogenetic animals [142] including in flies [4,58,112,114,115,149–151], cockroaches [117,118], stick insects [5], beetles [152], grasshoppers [153,154], moths [5], shrimp [5,155], crayfish [82], tardigrades [156], nematodes [90,123], planarians [157], rotifers [95], snails [5], salamanders [143] and fishes [158,159]. It seems that in many cases polyploidy can be a consequence of the method of becoming parthenogenetic, since hybrid parthenogens are often polyploid [102,160,161]. Polyploidy may result from incompatibilities between the two hybridizing genomes and may be selected for since polyploids probably have an increased adaptive potential in small populations owing to higher gene copy numbers [142]. Approximately two thirds of all polyploid animals are parthenogenetic [142] and polyploidy is associated with parthenogenetic reproduction in invertebrates and species hybridization in vertebrates [160]. It has been proposed that the increased genome copy number of these polyploid species may offer protection to the genome in lieu of recombination [6,162]. There are no *Drosophila* that are true breeding polyploids and only 14 polyploid dipterans (table 1). It appears that classical polypody is not common in parthenogenetic dipterans, although there is another phenomenon found in some dipteran families where there are germline restricted ploidy changes and these families also have a high incidence of parthenogenesis [163]. More species need to have their genome content examined to develop an accurate estimate of polyploid in parthenogenetic dipterans.

#### (iii) Mosaicism in facultative parthenogenesis

The formation of ploidy mosaics during parthenogenesis is a rare genomic abnormality that has been observed in *Drosophila* [4] (figure 3*d*). Similarly rare are fusions of the three haploid polar bodies to form a non-true breeding polyploid [4,63]. Mosaicism is predicted to be extremely rare [164] but has been documented in sexually reproducing frogs [165], turtles [145], lizards [110], birds [132,166] and humans [167]. It has also been found in parthenogenetic locusts [168], fishes [109,169,170] and turkeys [171]. As the phenomenon is difficult to detect, it might have a higher prevalence than described [164]. Whether such mosaicism results from non-disjunction, chimerism, or gamete duplication errors is not clear [8]. In *Drosophila*, it probably arises through polar body fusions in the early divisions, which would account for the 2n/3n mosaics that are usually observed.

## 6. Future perspectives

Many dipteran parthenogens are medically, economically and ecologically important species, and therefore understanding how and why parthenogenesis occurs in this order is key to understanding how these animals reproduce. A future understanding of parthenogenesis will require focus on its genetic causes to determine if the genes that have been found to cause parthenogenesis might be involved in the evolution of parthenogenesis in other dipteran species. Only when the genetic causes have been identified can the underlying mechanism begin to be elucidated and can we then begin to understand how and why parthenogenesis has repeatedly evolved from sexual reproduction. Parthenogenesis is a fundamental biological phenomenon that occurs in nearly every phylum of animals and demands our understanding if not simply for curiosity about life itself, then to determine whether human activity could be having an impact upon the evolution of parthenogenetic animals.

## Data Availability

The data are provided in the electronic supplementary material [172].
